# Comparison of aggregate and individual participant data approaches to meta-analysis of randomised trials: An observational study

**DOI:** 10.1371/journal.pmed.1003019

**Published:** 2020-01-31

**Authors:** Jayne F. Tierney, David J. Fisher, Sarah Burdett, Lesley A. Stewart, Mahesh K. B. Parmar

**Affiliations:** 1 MRC Clinical Trials Unit at UCL, Institute of Clinical Trials and Methodology, University College London, London, United Kingdom; 2 Centre for Reviews and Dissemination, University of York, York, United Kingdom; University of Pittsburgh, UNITED STATES

## Abstract

**Background:**

It remains unclear when standard systematic reviews and meta-analyses that rely on published aggregate data (AD) can provide robust clinical conclusions. We aimed to compare the results from a large cohort of systematic reviews and meta-analyses based on individual participant data (IPD) with meta-analyses of published AD, to establish when the latter are most likely to be reliable and when the IPD approach might be required.

**Methods and findings:**

We used 18 cancer systematic reviews that included IPD meta-analyses: all of those completed and published by the Meta-analysis Group of the MRC Clinical Trials Unit from 1991 to 2010. We extracted or estimated hazard ratios (HRs) and standard errors (SEs) for survival from trial reports and compared these with IPD equivalents at both the trial and meta-analysis level. We also extracted or estimated the number of events. We used paired *t* tests to assess whether HRs and SEs from published AD differed on average from those from IPD. We assessed agreement, and whether this was associated with trial or meta-analysis characteristics, using the approach of Bland and Altman. The 18 systematic reviews comprised 238 unique trials or trial comparisons, including 37,082 participants. A HR and SE could be generated for 127 trials, representing 53% of the trials and approximately 79% of eligible participants. On average, trial HRs derived from published AD were slightly more in favour of the research interventions than those from IPD (HR_AD_ to HR_IPD_ ratio = 0.95, *p =* 0.007), but the limits of agreement show that for individual trials, the HRs could deviate substantially. These limits narrowed with an increasing number of participants (*p <* 0.001) or a greater number (*p <* 0.001) or proportion (*p <* 0.001) of events in the AD. On average, meta-analysis HRs from published AD slightly tended to favour the research interventions whether based on fixed-effect (HR_AD_ to HR_IPD_ ratio = 0.97, *p =* 0.088) or random-effects (HR_AD_ to HR_IPD_ ratio = 0.96, *p =* 0.044) models, but the limits of agreement show that for individual meta-analyses, agreement was much more variable. These limits tended to narrow with an increasing number (*p =* 0.077) or proportion of events (*p =* 0.11) in the AD. However, even when the information size of the AD was large, individual meta-analysis HRs could still differ from their IPD equivalents by a relative 10% in favour of the research intervention to 5% in favour of control. We utilised the results to construct a decision tree for assessing whether an AD meta-analysis includes sufficient information, and when estimates of effects are most likely to be reliable. A lack of power at the meta-analysis level may have prevented us identifying additional factors associated with the reliability of AD meta-analyses, and we cannot be sure that our results are generalisable to all outcomes and effect measures.

**Conclusions:**

In this study we found that HRs from published AD were most likely to agree with those from IPD when the information size was large. Based on these findings, we provide guidance for determining systematically when standard AD meta-analysis will likely generate robust clinical conclusions, and when the IPD approach will add considerable value.

## Abstract 2

Jayne Tierney and colleagues compare findings of meta-analyses based on aggregate or individual patient data from a large set of oncology trials.

## Author summary

**Why was this study done?:**

- Most standard systematic reviews and meta-analyses of the effects of interventions are based on aggregate data (AD) extracted from trial publications.
- It is not clear when such AD meta-analyses provide reliable estimates of intervention effects.
- It is also not clear when the collection of more detailed individual participant data (IPD) is needed.

**What did the researchers do and find?:**

- Based on 18 cancer systematic reviews, we compared trial and meta-analysis results based on IPD with those based on AD.
- Results from AD were most likely to agree with those from IPD when the number of participants or events (absolute information size) and the proportion of participants or events available from the AD relative to the IPD (relative information size) were large.
- Based on findings from this study, we provide guidance on assessing when AD meta-analysis will likely lead to robust clinical conclusions, and when the IPD approach might add considerable value.

**What do these findings mean?:**

- If the absolute information size is small, AD meta-analysis results will be unreliable, and there will be little value in collecting IPD unless it will lead to a considerable increase in information.
- If the absolute information size is sufficient, but the relative information size small, AD meta-analysis results will be unreliable, and more AD and/or IPD will be needed.
- If both the absolute and relative information size are large, AD meta-analysis results will most likely be reliable, and the collection of IPD only useful if more detailed analyses are required.
- Our analyses may have lacked power to identify additional factors that might affect the reliability of AD meta-analyses, and we cannot be sure that our results are applicable to all outcomes and effect measures.

## Introduction

It remains unclear when standard systematic reviews and meta-analyses of published aggregate data (AD) are reliable enough to form robust clinical conclusions, and consequently when the ‘gold standard’ individual participant data (IPD) approach might be required. Most standard reviews continue to rely on published AD [1,2], and if some eligible trials are unpublished, or reported trial analyses are based on a subset of participants or outcomes, then information may be limited, and AD meta-analyses will be at risk of reporting biases [3]. There are additional considerations for AD meta-analyses evaluating the effects of interventions on time-to-event outcomes, which are frequently based on hazard ratios (HRs), either derived directly from trial publications, or estimated indirectly from published statistics or from data extracted from Kaplan–Meier (KM) curves [4–6]. Inevitably, each of these methods requires stronger and more assumptions, which, together with varying lengths of follow-up, could have repercussions for the reliability of the results.

The collection of IPD can help circumvent publication and other reporting biases associated with AD, provided data on unpublished trials and all (or most) participants and outcomes are obtained, and, if relevant, follow-up is extended beyond the time point of the trial publication [7–10]. Also, IPD enable more complex or detailed analyses, such as the investigation of whether intervention effects vary by participant characteristics [11]. However, it remains unclear whether the IPD approach is always needed for the reliable evaluation of the overall effects, and because these projects can take many years to complete, results may not be sufficiently timely. Moreover, the IPD approach may not be feasible, owing to the expertise and resources required [7,8] or to difficulties obtaining the necessary data. Hence, patients, clinicians, and policy makers will continue to rely on standard AD meta-analyses.

While some guidance is available to help reviewers gauge when AD might suffice and when IPD might add value [8,12], it is not backed by empirical evidence. A large systematic review of published AD versus IPD meta-analyses found that conclusions were often similar, but the comparisons could only be made on the basis of statistical significance [13]. For meta-analyses of published time-to-event outcomes, individual case studies have shown that they can produce effects that are larger than, smaller than, or similar to their IPD equivalents [14–23]. Bria et al. [24] compared effect estimates (HRs) from a cohort of AD meta-analyses with IPD equivalents and concluded that they gave very similar results. However, each AD meta-analysis had to include at least 90% of eligible participants and was compared to an IPD meta-analysis of the same set of trials, which may have minimised differences and is perhaps an unrealistic comparison of the 2 approaches. Moreover, both reviews [13,24] included multiple outcomes from the same meta-analyses, marring interpretation. Here, for a single outcome, we compare the results from a large cohort of cancer systematic reviews and meta-analyses based on IPD, with the best meta-analyses of published AD possible at the time these were completed, to establish when the latter are most likely to be reliable, and when the IPD approach might be required.

## Methods

The study did not follow a protocol or pre-specified plan. We reported the study according to the STROBE checklist.

### Data collection

We used a cohort of 18 cancer systematic reviews that included IPD meta-analyses: all of those completed and published by the Meta-analysis Group of the MRC Clinical Trials Unit at University College London over a 20-year period (1991 to 2010) [25–36], including updates where relevant. Each IPD review included a comprehensive search for all eligible trials, irrespective of publication status. Thus, at the time point each IPD meta-analysis was completed, we could ascertain which trials were published and include them in the related AD meta-analysis. This ensured that we were comparing each IPD meta-analysis with a meta-analysis of the published data available at that time. We used the corresponding publications for extraction of AD, and if a trial was reported in multiple publications, we used the one with the most up-to-date or complete information. Although a variety of research and control interventions were used, overall survival was the primary outcome in all of the meta-analyses, and the HR was the effect measure, so these are used as the basis for all our comparisons.

One author (JFT, SB, or DJF) independently extracted all data relevant to the derivation of the HR for the effect of treatment on overall survival and the associated standard error (SE) of its natural logarithm [4,6], and these data were crosschecked by another author. These data included reported HRs and SEs, confidence intervals and *p*-values, numbers of participants randomised and analysed, and numbers of events. If KM curves were available, we also extracted survival probabilities across a series of time intervals and the related numbers at risk [5,6], or the actual or estimated [4,6] minimum and maximum follow-up, to estimate HRs and SEs [4–6]. One author (JFT) reviewed all KM curve estimates to ensure a consistent approach to deciding the number and size of these intervals.

### Estimating HRs from published AD

We estimated the HRs and SEs using all possible methods [4–6], but preferentially used estimates calculated directly from the reported observed and expected events or the hazard rates for the research intervention and control groups [4,6]. If this was not possible, we used HRs and SEs estimated indirectly using a published log-rank, Mantel–Haenszel, or Cox *p*-value, and either the associated confidence interval or the number of events, provided the confidence intervals and *p*-values were given to at least 2 significant figures [4]. Finally, in the absence of these statistics, we used HRs and SEs derived from KM curves [4,6]. This meant we used the best possible estimate of each trial HR.

We matched each AD meta-analysis to the relevant IPD meta-analysis in terms of both the intervention comparisons and the analyses. Thus, if treatment effects were reported by participant subgroup, the subgroup HRs and SEs were combined using a fixed-effect inverse-variance meta-analysis to provide an appropriate AD estimate for the whole trial or treatment comparison. For a small number of 3-arm trials, we combined very similar treatment arms to provide a single estimate of treatment versus control. Whilst not best practice, we wanted to replicate the original analyses. For multi-arm trials with treatment comparisons that were eligible for different meta-analyses or a single treatment comparison that was eligible for more than 1 meta-analysis, estimates for the individual comparisons were included as appropriate. However, trials or treatment comparisons were not used more than once in the trial-level comparisons of HRs from AD and IPD.

### Statistical methods for comparing HRs from AD and IPD

We compared HRs and SEs derived from AD and IPD both at the trial level and meta-analysis level. At the trial level, we included all trials with both an AD and an IPD result. The meta-analyses were based on all available published AD and all available IPD, thus representing the best possible AD and IPD estimates available at the time the IPD meta-analysis was published. The IPD meta-analysis estimates were derived from the original IPD projects using 2-stage fixed-effect inverse-variance models, with trial-level HRs and SEs derived using Cox regression. We also performed sensitivity analyses using the DerSimonian and Laird random-effects model [37–39].

All data included in these analyses were aggregate in nature, whether derived from trial publications or from the original analyses of anonymised participant data, and therefore ethical approval was not required.

Estimates were compared on the log scale throughout, because the log HR is approximately normally distributed. However, we present the differences between log HRs from AD and IPD as back-transformed ratios of the AD HRs to the IPD HRs (i.e., the HR_AD_ to HR_IPD_ ratio). Differences between log SEs were also ‘back-transformed’ so that they are always greater than 0 and interpretable as relative percentage changes [40].

We used paired *t* tests to assess whether (log) HRs and SEs from AD differed on average from their IPD equivalents, recognising that the statistical significance of these tests relates to the amount of data available. More pertinently, we assessed agreement between HR and SE estimates from AD and IPD using the approach of Bland and Altman [40–42]_._ This involves plotting the differences between the AD and IPD estimates against their average, along with 95% ‘limits of agreement’ (defined as mean ± 1.96 × standard deviation), which represent a range within which most differences are expected to lie. Wide limits suggest poor agreement, although note they are not 95% confidence intervals and do not test a statistical hypothesis. At the trial level, we also used ANOVA to investigate whether the estimation method (direct, indirect, or KM curve) influenced the extent of agreement.

The Bland–Altman method also allowed us to examine whether agreement was associated with trial or meta-analysis characteristics. This involved plotting the differences between the AD and IPD log HRs against each characteristic and testing for a non-zero regression slope for the average agreement and for non-constant limits of agreement [40]. As described above, we initially plotted these differences against their averages, thus testing whether agreement improves or worsens with increasing size of the estimates [42]. We then went on to examine whether agreement was associated with the number of trials, participants, and events in the AD meta-analysis, as well as the proportion of trials, participants, and events in the AD meta-analysis relative to the IPD analysis. Regression slopes were reported as standardised beta coefficients.

Subsequently, we also used sensitivity analyses to assess whether agreement at the meta-analysis level might be improved by excluding trials where the reported analyses were at potential risk of bias [43] from incomplete outcome data or had limited or imbalanced follow-up. Pre-specified criteria were mutually agreed and applied independently by 2 authors (DJF and SB, or DJF and JFT). We considered trials that excluded greater than 10% of participants overall or that had a greater than 10% imbalance in patient exclusion by arm to be at potential risk of bias from incomplete outcome data [44]. Trials in which more than half of participants were estimated to have been censored prior to what would be considered an appropriate follow-up time for the site and stage of cancer (Table 1) were considered to have insufficient follow-up. We classified these based on the reported KM curves and extracted or estimated levels of censoring. Note that only trials judged to be at low risk of bias in terms of randomisation sequence generation and allocation concealment (based on information supplied by investigators and checking of the IPD) were included in our IPD meta-analyses.

**Table 1:** Characteristics of IPD and AD meta-analyses, and the methods used to obtain HRs for each (ordered by the degree of disagreement [Fig 5]).

**Meta-analysis**		**Total eligible**		Relative information size of the IPD meta-analysis (percent of total eligible)			Relative information size of the AD meta-analysis (percent of total eligible)			Number of trials in AD meta-analysis for which each method of estimating trial HR was used			Desired FU time (months)^†^	Survival at desired FU time (percent)^‡^
Name	Setting	Trials	Participants*	Trials	Participants	Events	Trials	Participants	Events**	HR	*p*-Value	KM curve		
Sarcoma [36]	High-risk, early sarcoma	17	1,605	14	1,568	709	10	1,120	306	1	2	7	60	60%
				(82%)	(98%)		(59%)	(70%)	(43%)					
Cervix 1 [32]	High-risk/locally advanced cervical cancer	21	2,242	18	2,074	1,084	11	1,969	815	1	2	8	48	48%
				(86%)	(93%)		(52%)	(88%)	(75%)					
Oesophagus [29]	Locally advanced oesophageal cancer	6	1,164	6	1,147	971	5	885	623	0	1	4	12	47%
				(100%)	(99%)		(83%)	(76%)	(64%)					
Cervix 2 [32]	High-risk/locally advanced cervical cancer	6	912	5	872	368	5	863	287	1	1	3	48	51%
				(83%)	(96%)		(83%)	(95%)	(78%)					
Lung PORT [35]	Operable NSCLC	12	2,418	11	2,343	1,511	7	1,274	789	3	1	3	24	58%
				(92%)	(97%)		(58%)	(53%)	(52%)					
Ovary 1 [27]	Advanced ovarian cancer	25	3,654	19	3,146	2,822	11	2,696	1,593	1	1	9	24	25%
				(76%)	(86%)		(44%)	(74%)	(56%)					
Lung 3 [33]	Locally advanced NSCLC	12	1,798	12	1,780	1,696	5	1,358	854	0	2	3	24	16%
				(100%)	(99%)		(42%)	(76%)	(50%)					
Bladder 1 [25]	Locally advanced bladder cancer	11	2,976	10	2,759	1,691	8	2,832	1,249	4	4	0	48	46%
				(91%)	(93%)		(73%)	(95%)	(74%)					
Bladder 2 [26]	Locally advanced bladder cancer	9	762	6	491	283	3	571	105	0	2	1	48	46%
				(67%)	(64%)		(33%)	(75%)	(37%)					
Ovary 5 [28]	Advanced ovarian cancer	13	2,381	12	2,220	1,745	6	2,168	864	3	1	2	24	48%
				(92%)	(93%)		(46%)	(91%)	(49%)					
Glioma [31]	High-grade glioma	19	3,767	12	3,004	2,659	11	3,316	2,225	1	1	9	6	71%
				(63%)	(80%)		(58%)	(88%)	(84%)					
Lung 2 [33]	Operable NSCLC	7	749	6	668	546	4	584	467	0	3	1	24	38%
				(86%)	(89%)		(57%)	(78%)	(85%)					
Lung 1 [33]	Operable NSCLC	8	1,394	8	1,394	614	3	584	280	1	2	0	60	49%
				(100%)	(100%)		(38%)	(42%)	(46%)					
Ovary 2 [27]	Advanced ovarian cancer	13	1,451	11	1,329	1,169	8	1,124	824	0	3	5	24	32%
				(85%)	(92%)		(62%)	(77%)	(70%)					
Ovary 4 [28]	Advanced ovarian cancer	9	1,102	9	1,095	894	5	1,014	668	0	2	3	24	41%
				(100%)	(99%)		(56%)	(92%)	(75%)					
Lung 4 [34]	Advanced NSCLC	18	3,349	15	2,714	2,533	12	3,219	2,212	4	3	5	12	19%
				(83%)	(81%)		(67%)	(96%)	(87%)					
Ovary 3 [28]	Advanced ovarian cancer	9	1,754	9	1,704	1,428	5	1,399	808	0	3	2	24	34%
				(100%)	(97%)		(56%)	(80%)	(57%)					
Cervix 3 [30]	High-risk/locally advanced cervical cancer	28	4,507	18	3,396	1,110	11	3,167	641	3	1	7	48	63%
				(64%)	(75%)		(39%)	(70%)	(58%)					
**Total**^¶^		**238**	**37,082**	**196**	**32,829**	**23,833**	**127**	**29,478**	**15,609**	**23**	**33**	**71**		
				**(82%)**	**(89%)**		**(53%)**	**(79%)**	**(65%)**					

*Exact numbers of eligible participants were not available for some (mostly small) unpublished trials, so this is our best estimate.

**Exact values where known, otherwise estimated by use of Formula 13 in Tierney et al. [6]. Percentages are for AD relative to IPD, since the total eligible is unknown.

^†^Chosen a priori by the authors of the present study, on the basis of the research question addressed by the review, in order to assess whether individual trials had an appropriate length of follow-up.

^‡^Estimated using all available IPD (i.e., from all trials) combined.

^¶^With duplicate trials removed.

AD, aggregate data; FU, follow-up; HR, hazard ratio; IPD, individual participant data; KM, Kaplan–Meier; NSCLC, non-small-cell lung cancer.

### A decision tree for assessing the reliability of AD meta-analyses

We utilised these results to construct a decision tree for assessing when AD meta-analyses are most likely to be reliable. As per reviewer comments, we have made this only as generalisable as the data allow.

## Results

### Feasibility of estimating HRs and associated SEs from published AD

The 18 systematic reviews included 243 trials, 5 of which were eligible for inclusion in 2 separate meta-analyses. Of the 238 unique trials, 33 (14%) were unpublished in any form, and 205 (86%) were published: 175 (74%) in peer-reviewed journals, 4 (2%) as book chapters, and 26 (11%) as abstracts in conference proceedings, with publication dates ranging from 1976 to 2005. HRs and SEs could be obtained or estimated from trial reports for 127 of the trials, representing 61% of published trials, 53% of all trials, and approximately 79% of eligible participants (Table 1). Of the remaining 78 trial reports, 49 (63%) did not include overall survival results (e.g., providing disease response or progression results instead) or presented survival results that could not be used to estimate a HR reliably (e.g., median survival [45] or survival rates); 8 (10%) included a KM curve, but with insufficient information to estimate censoring; 15 (19%) presented survival results, but not for the specific treatment comparison and/or data sample of interest; and 6 (8%) reports could not be accessed.

We obtained HR and SE estimates from IPD for 196 (82%) of trials, representing 89% of randomised participants (Table 1). As well as being able to include trials that had not been published, and trials that had not been reported in sufficient detail, we were also able to obtain additional participants that had been excluded from published analyses and additional events arising from updated follow-up.

The best available method for estimating HRs from published AD was direct extraction or calculation for 23 trials (18%), from a *p*-value for 31 trials (24%), and from a KM curve for 73 trials (57%; Table 1). For the SE, the best available method was direct extraction for 1 trial, from a confidence interval for 17 trials (13%), from the number of events for 58 trials (46%), and from a KM curve for 51 trials (40%). Where estimation from a KM curve was the best available method, the associated numbers at risk were reported for only 4 trials, so the minimum and maximum follow-up was used by default to estimate censoring [4].

### Reliability of trial HRs and SEs estimated from published AD

**Fig 1:**
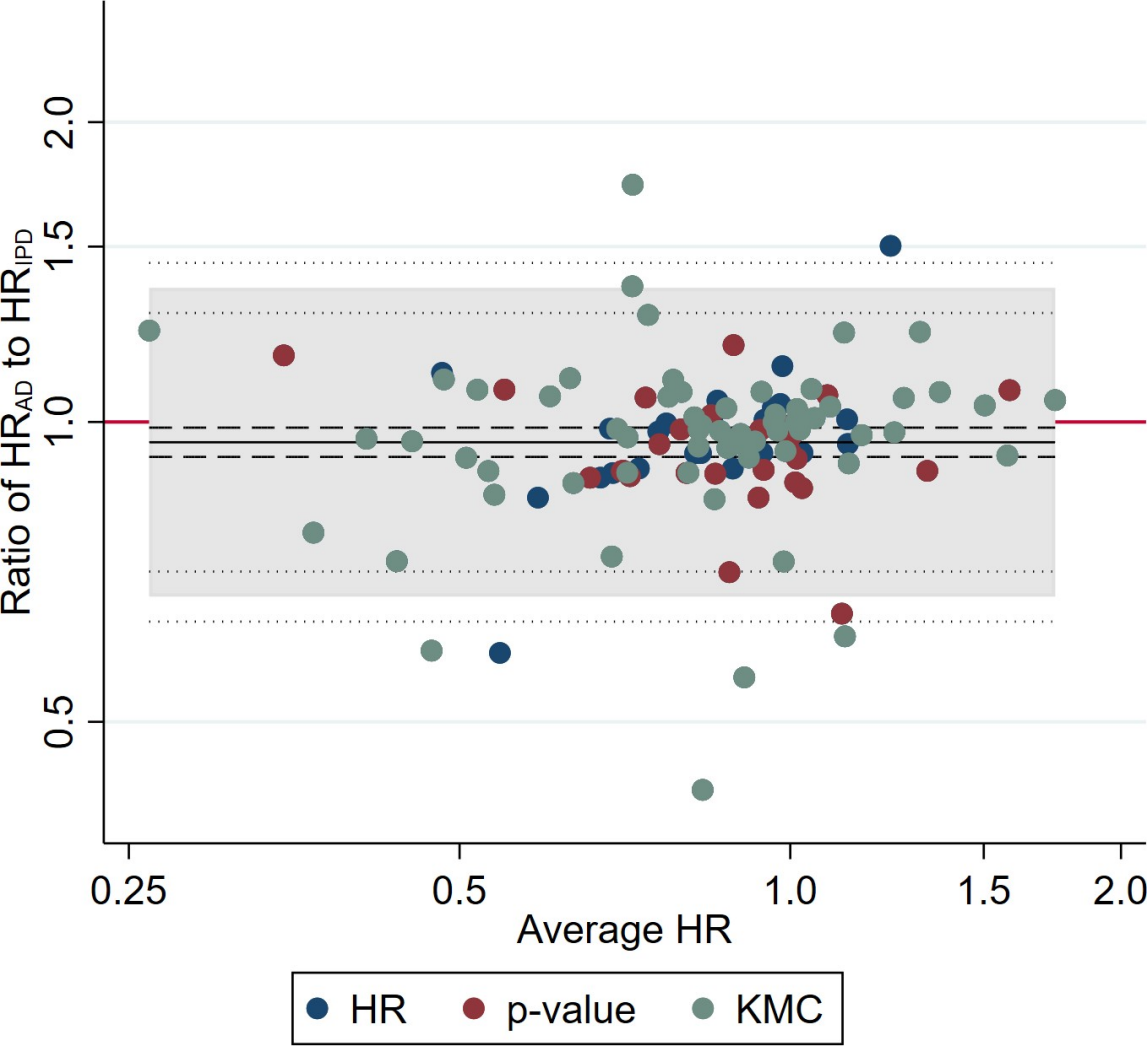
Comparison of trial HRs from AD versus IPD. Bland–Altman plot showing how the ratio of the HR from AD to the HR from IPD varies with the average HR (i.e., the geometric mean of the 2 HR estimates). The red horizontal line represents no difference (i.e., a ratio of 1). The shaded area represents the 95% Bland–Altman limits of agreement. Dashed and dotted lines represent statistical precision around the average ratio and the limits of agreement, respectively. Individual data points are distinguished by whether the AD estimate was derived directly from a reported HR, indirectly from a reported *p*-value and associated information, or indirectly from a Kaplan–Meier curve [6]. AD, aggregate data; HR, hazard ratio; IPD, individual participant data; KMC, Kaplan–Meier curve.

Among the 114 trials with estimates available from both AD and IPD, trial HRs derived from AD were on average slightly more in favour of the research intervention than those from IPD (HR_AD_ to HR_IPD_ ratio = 0.95, 95% CI 0.92 to 0.99, paired *t* test *p =* 0.007). However, the wide Bland–Altman limits of agreement (Fig 1) show that for any individual trial, HRs derived from AD could deviate from those derived from IPD by around a relative 30% in favour of either the research (HR_AD_ to HR_IPD_ ratio = 0.67) or control intervention (HR_AD_ to HR_IPD_ ratio = 1.36).

**Fig 2:**
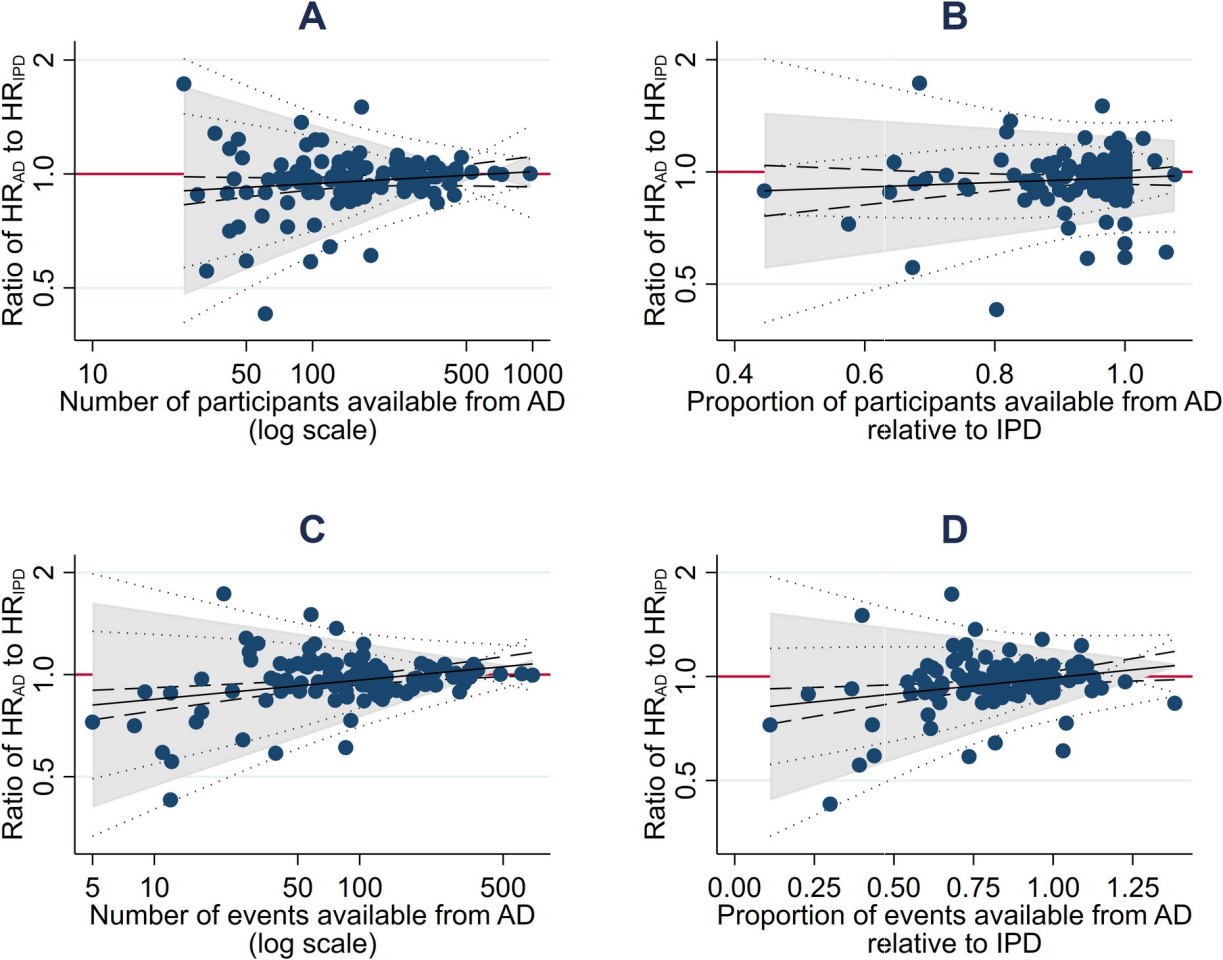
Potential predictors of the extent of agreement between trial HRs from AD and IPD. Bland–Altman plots showing how the ratio of the HR from AD to the HR from IPD varies according to the number of participants (A) and events (C) available from AD, and the proportion of participants (B) and events (D) available from AD relative to IPD. The red horizontal lines represent no difference (i.e., a ratio of 1). The shaded areas represent the 95% Bland–Altman limits of agreement, with fitted linear dependence upon the value of the covariate. Dashed and dotted lines represent statistical precision around the average ratios and the limits of agreement, respectively. AD, aggregate data; HR, hazard ratio; IPD, individual participant data.

There was no clear evidence that that agreement was associated with the size of effect (standardised β = +0.08, *p =* 0.39) or the estimation method (F statistic on 2 and 111 degrees of freedom = 0.26, *p =* 0.77; Fig 1). Also, there was no good evidence that agreement was related to the number (standardised β = +0.13, *p =* 0.17) or proportion (standardised β = −0.09, *p =* 0.36) of participants represented by the AD relative to IPD, but the limits of agreement did narrow as the absolute number of participants increased (standardised β = −0.45, *p <* 0.001). Moreover, average agreement improved (standardised β = +0.30 and +0.25, *p =* 0.001 and *p =* 0.009, respectively), and the limits of agreement narrowed (standardised β = −0.44 and −0.31, *p <* 0.001 and *p <* 0.001, respectively), as the absolute and relative number of events in the AD relative to the IPD increased (Fig 2).

Individual trial SEs based on AD were larger than those based on IPD (average percentage change = +12%, 95% CI +8% to +16%, *p <* 0.001, Bland–Altman 95% limits of agreement = −20% to +57%), which was more pronounced as the average SE increased (standardised β = +0.44, *p <* 0.001). After adjusting for this, agreement was also associated with a greater proportion of participants (standardised β = −0.15, *p =* 0.082) and number or proportion of events (standardised β = −4.55 and −0.88, respectively, *p <* 0.001 for both) being included in the AD analysis relative to the IPD analysis.

### Reliability of meta-analyses of HRs and SEs estimated from published AD

IPD were typically available for a high proportion of eligible trials (65% to 100%) and participants (75% to 100%; Table 1), with most including in excess of 85% of those eligible. While the AD meta-analyses tended to include a smaller proportion of eligible trials (33% to 83%; Table 1), often they still included a high proportion of eligible participants (42% to 96%; Table 1) relative to the IPD meta-analyses, but not necessarily such a high proportion of events (e.g., Sarcoma, Bladder 2, Ovary 5; Table 1).

**Fig 3:**
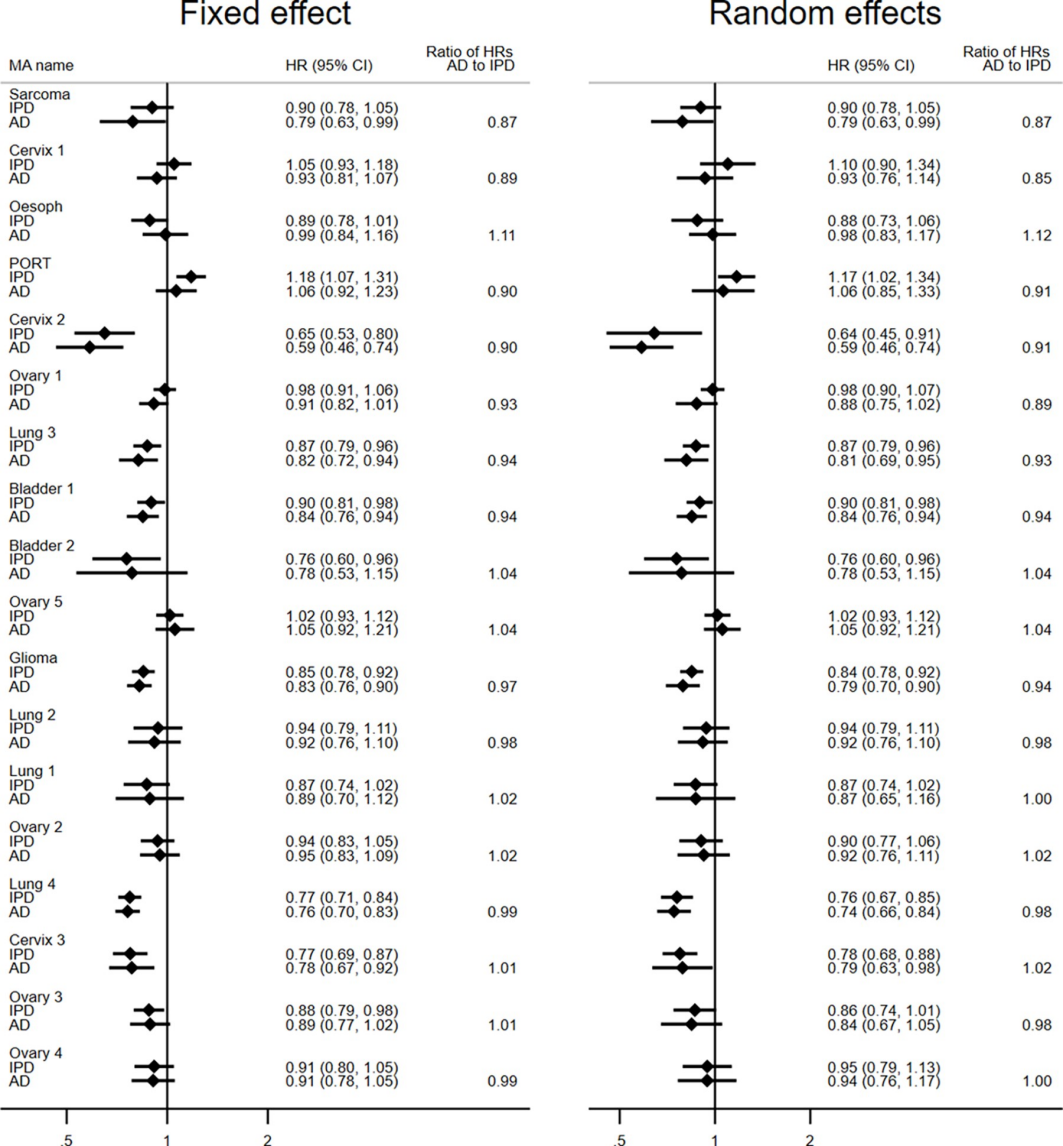
Forest plot of meta-analysis HRs and 95% confidence intervals from AD and IPD. Each filled diamond denotes the HR for AD or IPD based on fixed-effect and random-effects meta-analyses, with the horizontal lines showing the 95% CIs. Comparisons are ordered by the degree of disagreement, i.e., the HR_AD_ to HR_IPD_ ratio, irrespective of direction. AD, aggregate data; HR, hazard ratio; IPD, individual participant data; MA, meta-analysis.

**Fig 4:**
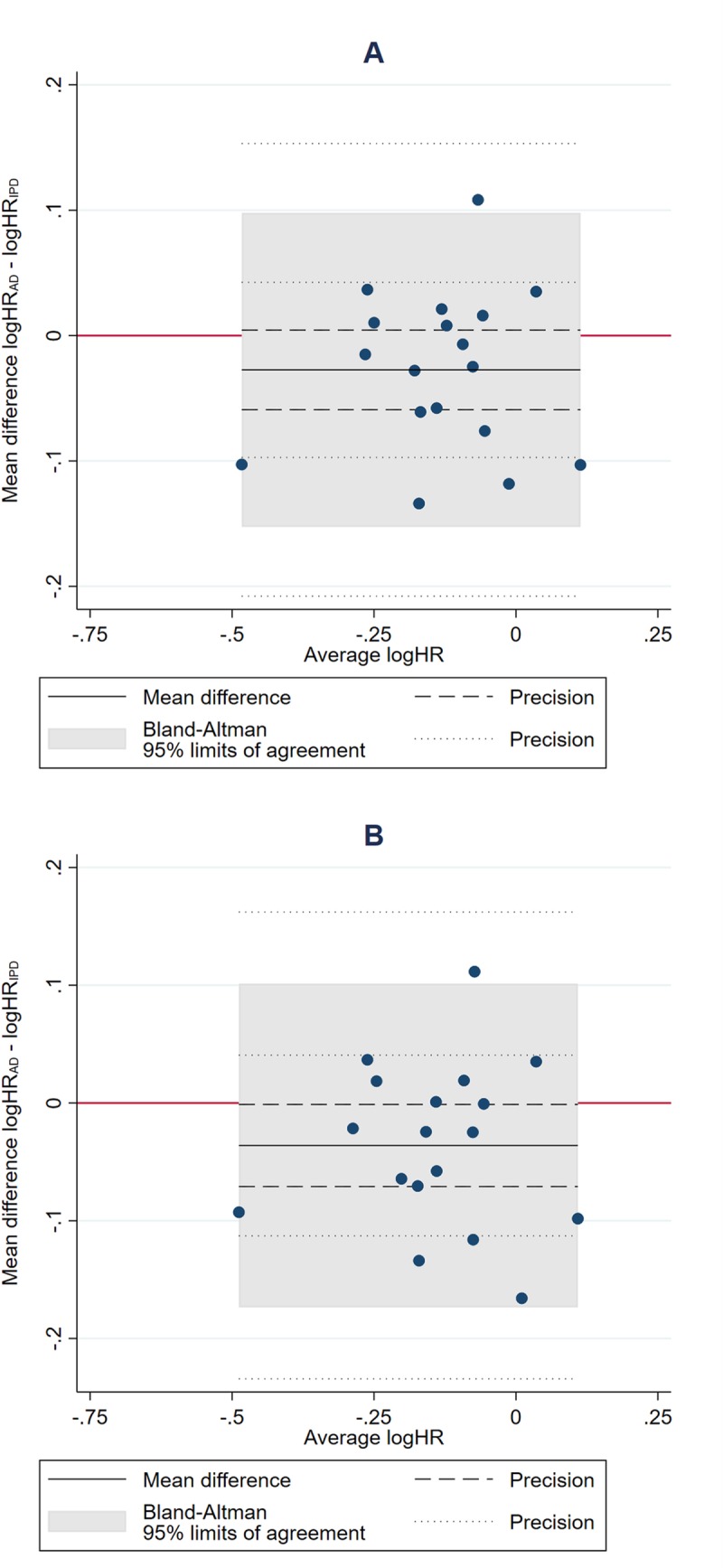
Comparison of meta-analysis HRs from AD versus IPD. Bland–Altman plots showing how the ratio of the HR from AD to the HR from IPD, as estimated by fixed-effect (A) and random-effects models (B), respectively, varies with the average HR (i.e., the geometric mean of the 2 HR estimates). The red horizontal line represents no difference (i.e., a ratio of 1). The shaded area represents the 95% Bland–Altman limits of agreement. Dashed and dotted lines represent statistical precision around the average ratio and the limits of agreement, respectively. AD, aggregate data; HR, hazard ratio; IPD, individual participant data.

Many HRs from AD and IPD meta-analyses were very similar (Fig 3), and, on average, meta-analyses from published AD were only slightly more likely to favour research interventions than those from IPD, irrespective of whether a fixed-effect (HR_AD_ to HR_IPD_ ratio = 0.97, 95% CI 0.94 to 1.00, paired *t* test *p =* 0.087) or random-effects (HR_AD_ to HR_IPD_ ratio = 0.96, 95% CI 0.93 to 0.99, paired *t* test *p =* 0.043; Fig 4) model was used. However, the Bland–Altman 95% limits of agreement suggest that an individual (fixed-effect) AD meta-analysis could deviate by up to around a relative 15% in favour of the research intervention (HR_AD_ to HR_IPD_ ratio = 0.86) to 10% (HR_AD_ to HR_IPD_ ratio = 1.10) in favour of control (Fig 4A). Findings were very similar with the random-effects model (Bland–Altman 95% limits of agreement for HR_AD_ to HR_IPD_ ratio = 0.84 to 1.11; Fig 4B).

**Fig 5:**
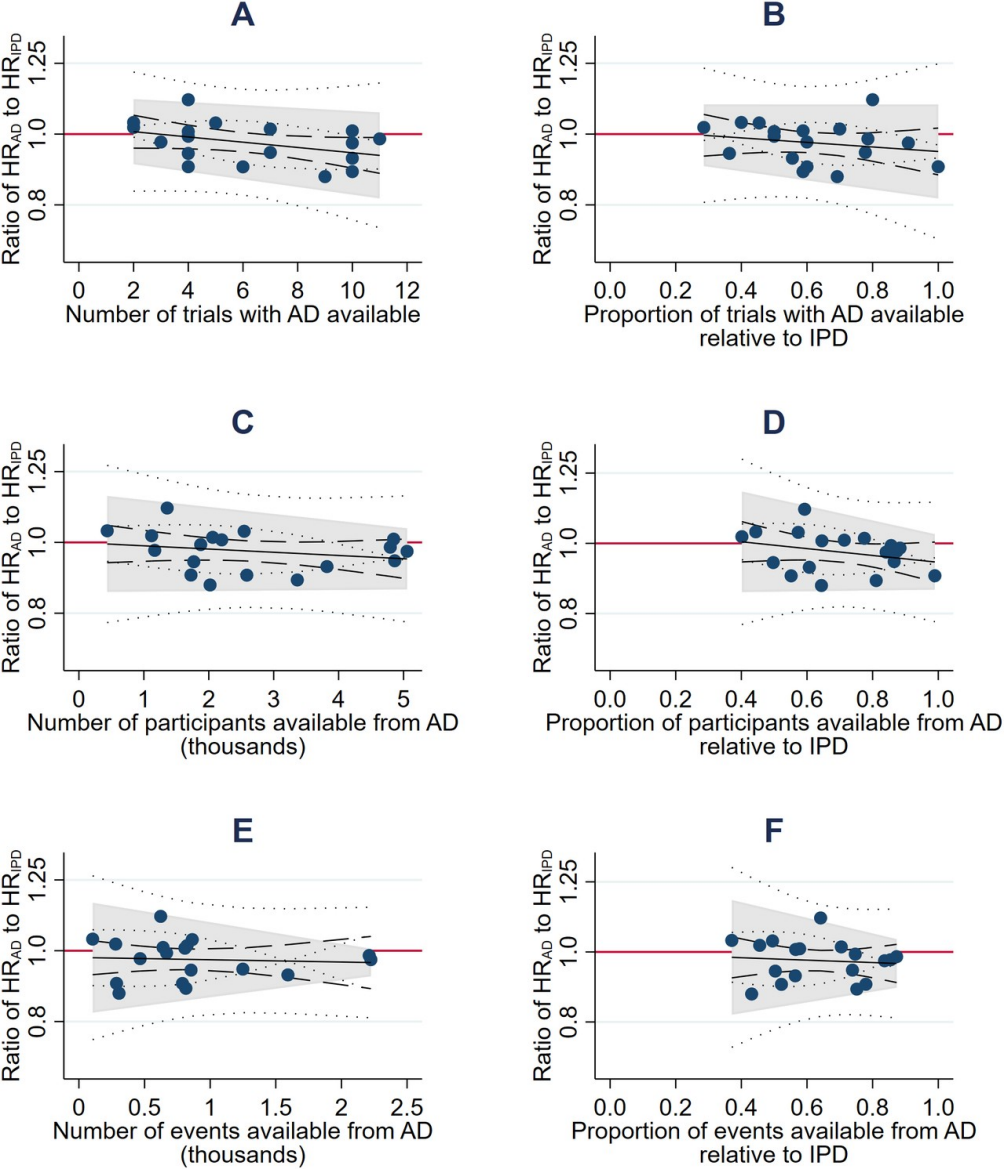
Potential predictors of the extent of agreement between (fixed-effect) meta-analysis HRs from AD and IPD. Bland–Altman plots showing how the ratio of the HR from AD to the HR from IPD varies according to the number of trials (A), participants (C), and events (E) available from AD, and the proportion of trials (B), patients (D), and events (F) available from AD relative to IPD. The red horizontal lines represent no difference (i.e., a ratio of 1). The shaded areas represent the 95% Bland–Altman limits of agreement, with fitted linear dependence upon the value of the covariate. Dashed and dotted lines represent statistical precision around the average ratios and the limits of agreement, respectively. AD, aggregate data; HR, hazard ratio; IPD, individual participant data.

Based on the fixed-effect model, there was no clear evidence that average agreement was associated with the average size of the HRs (standardised β = +0.06, *p =* 0.82; Fig 5A), the number (standardised β = −0.40, *p =* 0.099) or proportion (standardised β = −0.21, *p =* 0.40) of eligible trials (Fig 5A and 5B), or the number (standardised β = −0.23, *p =* 0.35) or proportion (standardised β = −0.29, *p =* 0.24) of eligible participants (Fig 5C and 5D). We also found no evidence that the limits of agreement narrowed when trials with published analyses at potential risk of bias from incomplete outcome data or that had limited or imbalanced follow-up were excluded (Table 2). There was some evidence that the limits of agreement became narrower as the total number of events (standardised β = −0.42, *p =* 0.079; Fig 5E), and, less clearly, the proportion of events (standardised β = −0.39, *p =* 0.11; Fig 5F), in the AD relative to IPD increased. However, even at the maximum proportion of events observed in this dataset (87% AD to IPD events), an AD meta-analysis might still differ from its IPD equivalent by around a relative 10% in favour of the research intervention (HR_AD_ to HR_IPD_ ratio = 0.90) to 5% in favour of control (HR_AD_ to HR_IPD_ ratio = 1.05). Statistical evidence for these associations was less clear under a random-effects model.

**Table 2:** Sensitivity analyses of the extent of agreement between meta-analysis HRs from AD versus IPD.

Sample	Fixed			Random		
	Average ratio HR_AD_ to HR_IPD_	95% limits of agreement*	*p*-Value**	Average ratio HR_AD_ to HR_IPD_	95% limits of agreement*	*p*-Value**
All trials in AD meta-analyses	0.97	0.86, 1.10	*p =* 0.088	0.96	0.84, 1.11	*p =* 0.044
Excluding trials at potential risk of bias from incomplete outcome data	0.95	0.86, 1.04	*p =* 0.23	0.96	0.84, 1.10	*p =* 0.023
Excluding trials with insufficient follow-up	0.97	0.86, 1.09	*p =* 0.056	0.91	0.62, 1.34	*p =* 0.069

*Calculated on the log scale using the method of Bland and Altman.

**From *t* tests of AD versus IPD log HRs.

AD, aggregate data; HR, hazard ratio; IPD, individual participant data.

Meta-analysis SEs were consistently larger with AD compared to IPD by an average of around 30% (e.g., fixed-effect 95% CI 18% to 35%; fixed-effect and random-effects *p <* 0.001), with wide Bland–Altman limits of agreement (e.g., fixed-effect 95% limits of agreement −3% to +63%). Not surprisingly, agreement improved when a greater proportion of trials (standardised β = −0.63, *p =* 0.005), participants (standardised β = −0.89, *p <* 0.001), and events (standardised β = −0.99, *p <* 0.001) were included in the AD meta-analysis. These associations all remained significant under a random-effects model.

### A decision tree for assessing the reliability of AD meta-analyses of HRs

**Fig 6:**
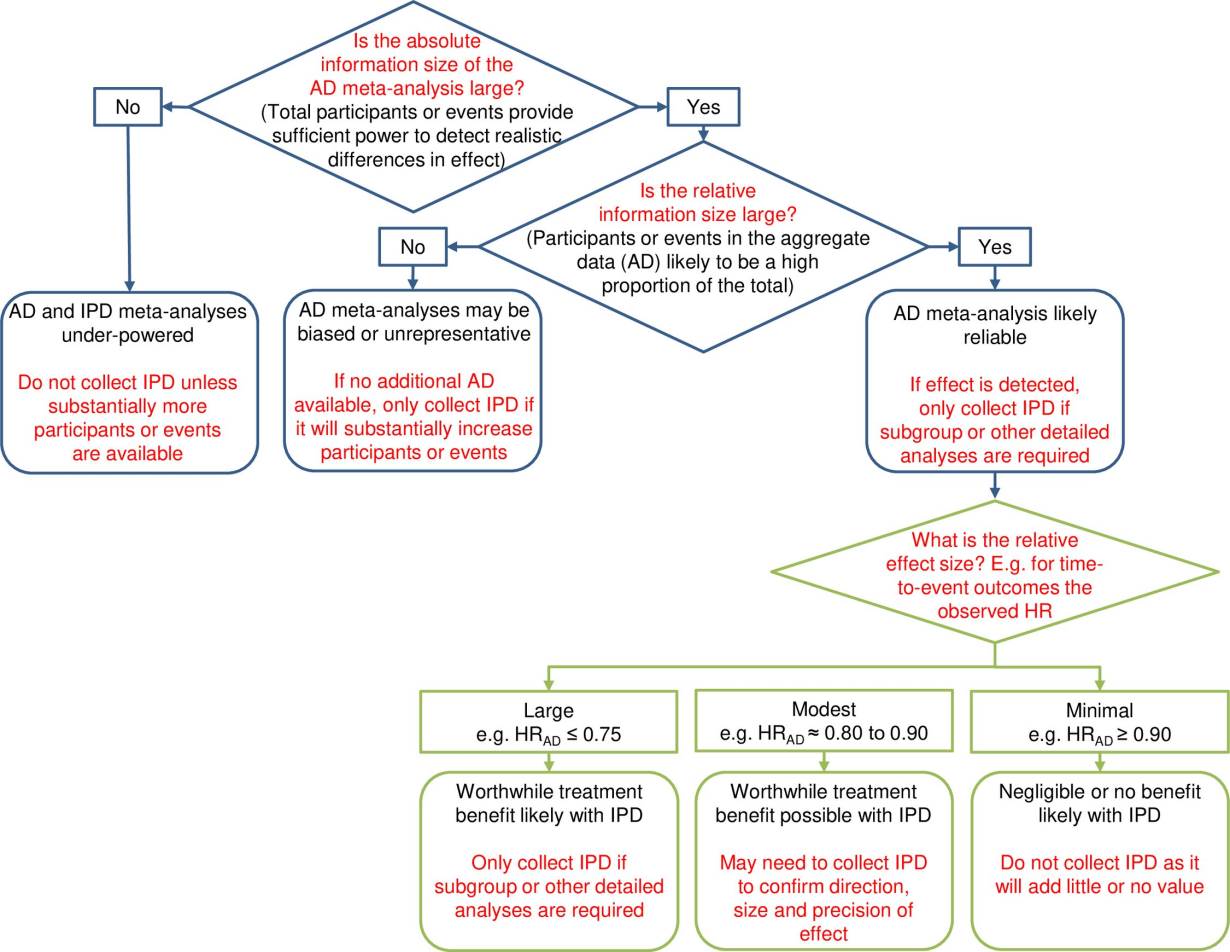
Decision tree for assessing when AD meta-analysis HRs are likely reliable and when the IPD approach might be required. AD, aggregate data; HR, hazard ratio; IPD, individual participant data.

Taking results at the trial and meta-analysis level together, HRs derived from published AD were most likely to concur with those from IPD when the overall number of participants or events (‘absolute information size’) was high, and also when the proportion of events included in the AD relative to the IPD (‘relative information size’) was high. Hence, ascertaining the absolute and relative information size of the available AD is a critical part of determining whether a meta-analysis of published HRs is sufficient for robust syntheses, and when IPD might be needed (Fig 6). Intuitively, establishing information size should also be a goal for AD meta-analyses of other outcomes and effect measures. For time-to-event outcomes and binary outcomes, information size will mostly relate to the number of participants and events, and for continuous outcomes, to the number of participants.

The starting point for assessing the absolute information size is to establish the total number of eligible participants and, if relevant/possible, the number of events. For accuracy, this assessment needs to be based on all trials whether published, unpublished, or ongoing, and the actual or projected accrual figures for each. If the absolute information size is small, an AD meta-analysis will lack power and be unreliable. Also, the collection of IPD will add little value unless it can bring about an increase in the number of participants or events (Fig 6).

If the absolute information size is deemed sufficient, but AD are only available for a small proportion of the eligible participants or the number of events is low, it follows that the relative information size will be small, and any AD estimate is likely to be unreliable. If further AD are not available, the collection of IPD could be very valuable in increasing the number of participants or events (Fig 6).

If the absolute information size is adequate, and AD are available for a large proportion of the eligible participants, and/or most events have already happened, the relative information size is likely to be large, and an AD meta-analysis is expected to be reliable. In this scenario, the collection of IPD would only be useful if an intervention effect has been detected and more detailed analyses are required.

Our results also suggest that there may still be uncertainty in the size and direction of effect, which could influence any decision to collect IPD. In particular, for time-to-event outcomes, we found that even if both the absolute and relative information size of an AD meta-analysis are large, an AD meta-analysis HR can still differ unpredictably from its IPD equivalent, by an approximate relative 10% in favour of the research interventions (HR_AD_ to HR_IPD_ ratio = 0.90) to 5% in favour of control (HR_AD_ to HR_IPD_ ratio = 1.05). By applying these limits to a plausible range of AD meta-analysis HRs (i.e., dividing them by 0.90 and 1.05), we can see how estimates might change when IPD are collected and what these would mean in absolute terms. This helps to gauge which observed HRs are most likely to be reliable (Table 3). For example, an observed HR ≤ 0.75 would translate mostly to sizeable potential IPD absolute benefits, and therefore a benefit is likely confirmed without the need for IPD (Table 3; Fig 6). For an observed AD meta-analysis HR of around 0.80 to 0.90, the potential IPD absolute effects would not necessarily be clinically worthwhile (Table 3). Hence, IPD might be needed to provide a greater degree of certainty about whether an effect exists, and its size and precision (Fig 6). Finally, with an observed AD meta-analysis HR ≥ 0.95, a lack of benefit is probably confirmed, and the collection of IPD would be difficult to justify (Table 3; Fig 6). Note that our example HR ranges purposefully leave gaps, reflecting regions where the reliability of AD and need for IPD may be context-specific and harder to judge (Table 3).

**Table 3:** Application of the Bland–Altman limits of agreement to a plausible range of AD meta-analysis HRs.

Observed AD meta-analysis HR	Potential IPD meta-analysis HR (after dividing by the limits of agreement: 0.90 and 1.05)	Observed control group survival band	Potential IPD meta-analysis absolute survival effects at a representative control group value
**0.70**	0.67 to 0.78	<10%	5% to 8%
		10%–19%	8% to 13%
		20%–49%	9% to 14%
		50%–69%	7% to 11%
		≥70%	5% to 7%
**0.75**	0.71 to 0.83	<10%	3% to 7%
		10%–19%	6% to 10%
		20%–49%	7% to 11%
		50%–69%	5% to 9%
		≥70%	3% to 6%
**0.80**	0.76 to 0.89	<10%	2% to 5%
		10%–19%	4% to 8%
		20%–49%	4% to 10%
		50%–69%	4% to 8%
		≥70%	2% to 5%
**0.85**	0.81 to 0.94	<10%	1% to 4%
		10%–19%	2% to 6%
		20%–49%	2% to 7%
		50%–69%	2% to 6%
		≥70%	1% to 4%
**0.90**	0.86 to 1.00	<10%	0% to 2%
		10%–19%	0% to 4%
		20%–49%	0% to 5%
		50%–69%	0% to 4%
		≥70%	0% to 3%
**0.95**	0.90 to 1.05	<10%	−1% to 1%
		10%–19%	−2% to 3%
		20%–49%	−2% to 3%
		50%–69	−2% to 3%
		≥70%	−1% to 2%

AD, aggregate data; HR, hazard ratio; IPD, individual participant data.

## Discussion

### Findings

We compared trial and meta-analysis HRs from published AD with those from IPD, and found they were most likely to agree when both the absolute and relative information size (number and proportion of events or participants) of the AD were large. However, the AD meta-analysis results could still differ from their IPD equivalents by up to a relative 10% in favour of the research interventions to 5% in favour of control. There was no clear evidence that agreement between meta-analysis HRs from AD and IPD was associated with the number or proportion of eligible trials or the number participants included in the AD analyses, or the method of estimating the HR. Furthermore, agreement was not improved by excluding trials with reported analyses that were potentially at risk of bias from incomplete outcome data or that had insufficient follow-up. These results have been used to construct a decision tree for determining when an AD meta-analysis might be sufficiently reliable and when the IPD approach might be required (Fig 6).

### Context

Our results support the assertion that in order for a meta-analysis to be reliable, the information size should be at least as large as an adequately powered trial [46]. Although there is greater interest now in estimating the (absolute) information size of meta-analyses [47–52], surprisingly little attention has been paid to explicitly quantifying the relative information size of an AD meta-analysis [48–51]. A comprehensive systematic review of published comparisons of AD and IPD meta-analyses did not find that agreement was associated with the information they contained (the number of trials or participants) [53], but without access to the primary studies, the authors could not investigate this more thoroughly, and, as stated previously, multiple outcomes from the same meta-analyses were included. However, the authors recommend that systematic reviewers conduct an AD meta-analysis first and carefully consider the potential benefits of an IPD meta-analysis [13], and our decision tree provides the means to do this.

Unlike previous studies [4], there was no strong indication that HRs estimated indirectly from KM curves were systematically biased, at either the trial or meta-analysis level. In fact, some AD meta-analyses that relied heavily on HRs estimated from KM curves were very similar to their IPD equivalents. Thus, if other survival statistics cannot be obtained, we encourage reviewers to include HRs estimated carefully from KM curves [6]. Although alternative weighting approaches [54] and digital methods to extract data from KM curves [55] have emerged, they do not necessarily improve HR estimates [55]. However, a HR may not always be the most appropriate effect measure, for example, if there are non-proportional hazards within 1 or more trials in a meta-analysis. Non-proportionality of hazards can be readily checked with IPD and alternative effect measures used if desired (e.g., Wei et al. [56]), but such checks are also possible with AD [57], if ‘IPD’ can be reconstructed from published KM curves [55].

### Strengths

To our knowledge, our study represents the largest systematic comparison of trial and meta-analysis HRs from AD and IPD, and is the first to reveal characteristics associated with the reliability of results based on published AD. Our findings are based on all cancer systematic reviews and meta-analyses of IPD conducted by the MRC Clinical Trials Unit at University College London over a 20-year period. By utilising a cohort of 18 reviews and 238 unique trials, we avoid the potential publication bias that might be associated with reviewing published comparisons of AD and IPD meta-analyses [13]. The sample is diverse in terms of the cancer and intervention types, number of trials and participants, availability of data, and mix of methods used to estimate the AD HRs (Table 1), which increases generalisability. From recent data [1], we estimate that approximately 1,200 oncology intervention reviews are published each year, which may be of variable quality, so we expect our findings to be of widespread use. IPD were collected for over 80% of eligible trials and nearly 90% of eligible participants, and often included updated follow-up. Thus, the included IPD meta-analyses provide a true ‘gold standard’ with which to compare the HRs derived from AD.

### Limitations

Our analyses may lack power at the meta-analysis level, which could have prevented us identifying additional factors associated with the reliability of AD meta-analyses based on HRs. Also, we cannot be sure that results from a cohort of cancer systematic reviews are entirely generalisable to other healthcare areas and outcomes, although they do emphasise that information size should be considered alongside the direction, precision, and consistency of effects, when appraising an AD meta-analysis. Only about half of the eligible trials were included in the AD meta-analyses, but these trials represented around 80% of participants, minimising the impact of selective outcome reporting bias [58] on our findings. However, we could only estimate a HR and SE for 61% of published eligible trials in our time window of 1991–2010, a situation that has likely improved since the publication of the CONSORT statement [59,60]. Thus, we would strongly encourage other custodians of multiple IPD meta-analyses to do similar comparisons and add to this body of evidence, particularly for other conditions, outcomes, and effect measures. In the meantime, it is worthwhile factoring a degree of uncertainty into the interpretation of any AD meta-analysis.

### Implications

Once the absolute and relative information size of an AD meta-analysis have been ascertained, our decision tree can be used to systematically assess whether it will likely suffice or if IPD might be required (Fig 6). If the absolute information size indicates that a meta-analysis will be clearly underpowered to assess the primary research question, we do not recommend the collection of IPD unless it would lead to a considerable increase in information, for example, as a result of further follow-up of the included trials or reinstatement of participants that were excluded from the published analyses. If an AD meta-analysis likely has power but the relative information size is small, the meta-analysis results are more likely to be biased or otherwise unreliable, and the collection of further AD should be prioritised, for example, from trials that are unpublished or published in insufficient detail. If this is not feasible, but the collection of IPD could bring about a substantial increase in the amount of information, this is where the approach could add considerable value. If the absolute and relative information size of the AD are both large, the results of an AD meta-analysis are most likely reliable, so if there is no evidence of an effect, there is little justification for going to the trouble of collecting IPD. Whereas, if an effect has been detected based on AD, there may be motivation to collect IPD in order to conduct subgroup or other detailed analyses and provide more nuanced results. The absolute and relative information size are also useful for anticipating when accumulating evidence from trials might be sufficient for reliable AD meta-analysis, using a prospective framework for adaptive meta-analysis (FAME) [48–51].

## Conclusions

In this study, we show how to determine systematically when standard AD meta-analysis will likely generate robust clinical conclusions, and when the IPD approach will add considerable value.

## Supporting information

S1 Checklist Completed STROBE checklist for the study. (DOCX)

S1 Data Summary data underlying the study analyses. (XLSX)

## Abbreviations

AD: aggregate data
HR: hazard ratio
IPD: individual participant data
KM: Kaplan–Meier
SE: standard error

## References

[1] Page MJ, Shamseer L, Altman DG, Tetzlaff J, Sampson M, Tricco AC, et al Epidemiology and reporting characteristics of systematic reviews of biomedical research: a cross-sectional study. PLoS Med. 2016;13(5):e1002028 10.1371/journal.pmed.1002028 27218655 PMC4878797

[2] Baudard M, Yavchitz A, Ravaud P, Perrodeau E, Boutron I. Impact of searching clinical trial registries in systematic reviews of pharmaceutical treatments: methodological systematic review and reanalysis of meta-analyses. BMJ. 2017;356:j448 10.1136/bmj.j448 28213479 PMC5421496

[3] Sterne JAC, Egger M, Moher D, Cochrane Bias Methods Group. Addressing reporting biases In: Higgins JPT, Green S, editors. Cochrane handbook for systematic reviews of interventions. Chichester (UK): John Wiley & Sons; 2008 pp. 297–333.

[4] Parmar MKB, Torri V, Stewart LA. Extracting summary statistics to perform meta-analyses of the published literature for survival endpoints. Stat Med. 1998;17:2815–34. 10.1002/(sici)1097-0258(19981230)17:24<2815::aid-sim110>3.0.co;2-8 9921604

[5] Williamson PR, Tudur Smith C, Hutton JL, Marson AG. Aggregate data meta-analysis with time-to-event outcomes. Stat Med. 2002;21:3337–51. 10.1002/sim.1303 12407676

[6] Tierney JF, Stewart LA, Ghersi D, Burdett S, Sydes MR. Practical methods for incorporating summary time-to-event data into meta-analysis. Trials. 2007;8(1):16. 17555582 10.1186/1745-6215-8-16 PMC1920534

[7] Stewart LA, Clarke MJ, Cochrane Working Party Group on Meta-analysis using Individual Patient Data. Practical methodology of meta-analyses (overviews) using updated individual patient data. Stat Med. 1995;14:2057–79. 10.1002/sim.4780141902 8552887

[8] Stewart LA, Tierney JF. To IPD or not to IPD? Advantages and disadvantages of systematic reviews using individual patient data. Eval Health Prof. 2002;25(1):76–97. 10.1177/0163278702025001006 11868447

[9] Stewart LA, Tierney JF, Clarke M, Cochrane Individual Patient Data Meta-analysis Methods Group. Reviews of individual patient data In: Higgins JPT, Green S, editors. Cochrane handbook for systematic reviews of interventions. Chichester (UK): John Wiley & Sons; 2008 pp. 547–58.

[10] Tierney JF, Vale CL, Riley R, Tudur Smith C, Stewart LA, Clarke M, et al Individual participant data (IPD) meta-analyses of randomised controlled trials: guidance on their use. PLoS Med. 2015;12(7):e1001855 10.1371/journal.pmed.1001855 26196287 PMC4510878

[11] Fisher DJ, Carpenter JR, Morris TP, Freeman SC, Tierney JF. Meta-analytical methods to identify who benefits most from treatments: daft, deluded, or deft approach? BMJ. 2017;356:j573 10.1136/bmj.j573 28258124 PMC5421441

[12] Tudur Smith C, Clarke M, Marson T, Riley R, Stewart L, Tierney J, et al A framework for deciding if individual participant data are likely to be worthwhile. Abstracts of the 23rd Cochrane Colloquium, Vienna, Austria, 3–7 October 2015. Cochrane Database Syst Rev. 2015;10(Suppl):RO 6.1.

[13] Tudur Smith C, Marcucci M, Nolan SJ, Iorio A, Sudell M, Riley R, et al Individual participant data meta-analyses compared with meta-analyses based on aggregate data. Cochrane Database Syst Rev. 2016;9:MR000007 10.1002/14651858.MR000007.pub3 27595791 PMC7125394

[14] Stewart LA, Parmar MKB. Meta-analysis of the literature or of individual patient data: is there a difference? Lancet. 1993;341:418–22. 10.1016/0140-6736(93)93004-k 8094183

[15] Pignon J-P, Arriagada R. Meta-analysis. Lancet. 1993;341(8850):964–5. 8096301

[16] Clarke M, Godwin J. Systematic reviews using individual patient data: a map for the minefields? Ann Oncol. 1998;9:827–33. 10.1023/a:1008468705492 9789604

[17] Ioannidis JP, Collier AC, Cooper DA, Corey L, Fiddian AP, Gazzard BG, et al Clinical efficacy of high-dose acyclovir in patients with human immunodeficiency virus infection: a meta-analysis of randomized individual patient data. J Infect Dis. 1998;178(2):349–59. 10.1086/515621 9697714

[18] Szczech LA, Berlin JA, Feldman HI. The effect of antilymphocyte induction therapy on renal allograft survival. A meta-analysis of individual patient-level data. Anti-Lymphocyte Antibody Induction Therapy Study Group. Ann Intern Med. 1998;128(10):817–26. 10.7326/0003-4819-128-10-199805150-00004 9599193

[19] Best L, Simmons P, Baughan C, Buchanan R, Davis C, Fentiman I, et al Palliative chemotherapy for advanced or metastatic colorectal cancer. Cochrane Database Syst Rev. 2000;2000:CD001545. 10.1002/14651858.CD001545 PMC7025779 10796809

[20] Williamson PR, Marson AG, Tudur C, Hutton JL, Chadwick D. Individual patient data meta-analysis of randomized anti-epileptic drug monotherapy trials. J Eval Clin Pract. 2000;6(2):205–14. 10.1046/j.1365-2753.2000.00236.x 10970014

[21] Duchateau L, Pignon J-P, Bijnens L, Bertin S, Bourhis J, Sylvester R. Individual patient-versus literature-based meta-analysis of survival data: time to event and event rate at a particular time can make a difference, an example based on head and neck cancer. Control Clin Trials. 2001;22(5):538–47. 10.1016/s0197-2456(01)00152-0 11578787

[22] Brouwer IA, Raitt MH, Dullemeijer C, Kraemer DF, Zock PL, Morris C, et al Effect of fish oil on ventricular tachyarrhythmia in three studies in patients with implantable cardioverter defibrillators. Eur Heart J. 2009;30(7):820–6. 10.1093/eurheartj/ehp003 19196720 PMC2663728

[23] Rejnmark L, Avenell A, Masud T, Anderson F, Meyer HE, Sanders KM, et al Vitamin D with calcium reduces mortality: patient level pooled analysis of 70,528 patients from eight major vitamin D trials. J Clin Endocrinol Metab. 2012;97(8):2670–81. 10.1210/jc.2011-3328 22605432 PMC3410276

[24] Bria E, Gralla RJ, Raftopoulos H, Sperduti I, Millela M, Cognetti F, et al Assessing two meta-analysis (MA) methods: individual patient data-based (IPD) versus literature-based abstracted data (AD) in 10 MA including 37,002 patients (pts)—are there differences of concern? J Clin Oncol. 2011;29(15 Suppl):Abstract 6054.

[25] Advanced Bladder Cancer (ABC) Meta-analysis Collaboration. Neoadjuvant chemotherapy in invasive bladder cancer: update of a systematic review and meta-analysis of individual patient data. Eur Urol. 2005;48(2):202–6. 10.1016/j.eururo.2005.04.006 15939524

[26] Advanced Bladder Cancer (ABC) Meta-analysis Collaboration. Adjuvant chemotherapy in invasive bladder cancer: a systematic review and meta-analysis of individual patient data. Eur Urol. 2005;48(2):189–201. 10.1016/j.eururo.2005.04.005 15939530

[27] Advanced Ovarian Cancer Trialists Group. Chemotherapy in advanced ovarian cancer: an overview of randomised clinical trials. BMJ. 1991;303:884–93. 10.1136/bmj.303.6807.884 1834291 PMC1671193

[28] Aabo K, Adams M, Adnitt P, Alberts DS, Athanazziou A, et al Chemotherapy in advanced ovarian cancer: four systematic meta-analyses of individual patient data from 37 randomized trials. Advanced Ovarian Cancer Trialists’ Group. Br J Cancer. 1998;78(11):1479–87. 10.1038/bjc.1998.710 9836481 PMC2063202

[29] Arnott SJ, Duncan W, Gignoux M, David GJ, Hansen HS, Launois B, et al Preoperative radiotherapy in esophageal carcinoma: a meta-analysis using individual patient data (Oesophageal Cancer Collaborative Group). Int J Radiat Oncol Biol Phys. 1998;41(3):579–83. 10.1016/s0360-3016(97)00569-5 9635705

[30] Chemoradiotherapy for Cervical Cancer Meta-Analysis Collaboration. Reducing uncertainties about the effects of chemoradiotherapy for cervical cancer: a systematic review and meta-analysis of individual patient data from 18 randomized trials. J Clin Oncol. 2008;26(35):5802–12. 10.1200/JCO.2008.16.4368 19001332 PMC2645100

[31] Glioma Meta-analysis Trialists (GMT) Group. Chemotherapy in adult high-grade glioma: a systematic review and meta-analysis of individual patient data from 12 randomised trials. Lancet. 2002;359(9311):1011–18. 10.1016/s0140-6736(02)08091-1 11937180

[32] Neoadjuvant Chemotherapy for Cervix Cancer Meta-analysis Collaboration. Neoadjuvant chemotherapy for locally advanced cervical cancer: a systematic review and meta-analysis of individual patient data from 21 randomised trials. Eur J Cancer. 2003;39(17):2470–86. 10.1016/s0959-8049(03)00425-8 14602133

[33] Non-small Cell Lung Cancer Collaborative Group. Chemotherapy in non-small cell lung cancer: a meta-analysis using updated data on individual patients from 52 randomised clinical trials. BMJ. 1995;311:899–909. 7580546 PMC2550915

[34] NSCLC Meta-Analyses Collaborative Group. Chemotherapy in addition to supportive care improves survival in advanced non-small-cell lung cancer: a systematic review and meta-analysis of individual patient data from 16 randomized controlled trials. J Clin Oncol. 2008;26(28):4617–25. 10.1200/JCO.2008.17.7162 18678835 PMC2653127

[35] PORT Meta-analysis Trialists Group. Postoperative radiotherapy in non-small-cell lung cancer: systematic review and meta-analysis of individual patient data from nine randomised controlled trials. Lancet. 1998;352:257–63. 9690404

[36] Sarcoma Meta-analysis Collaboration. Adjuvant chemotherapy for localised resectable soft-tissue sarcoma of adults: meta-analysis of individual patient data. Lancet. 1997;350:1647–54. 9400508

[37] DerSimonian R, Laird N. Meta-analysis in clinical trials. Control Clin Trials. 1986;7:177–88. 10.1016/0197-2456(86)90046-2 3802833

[38] Röver C, Knapp G, Friede T. Hartung-Knapp-Sidik-Jonkman approach and its modification for random-effects meta-analysis with few studies. BMC Med Res Methodol. 2015;15:99 10.1186/s12874-015-0091-1 26573817 PMC4647507

[39] Hardy RJ, Thompson SG. A likelihood approach to meta-analysis with random effects. Stat Med. 1996;15(6):619–29. 10.1002/(SICI)1097-0258(19960330)15:6<619::AID-SIM188>3.0.CO;2-A 8731004

[40] Bland JM, Altman DG. Measuring agreement in method comparison studies. Stat Methods Med Res. 1999;8(2):135–60. 10.1177/096228029900800204 10501650

[41] Bland JM, Altman DG. Statistical methods for assessing agreement between two methods of clinical measurement. Lancet. 1986;1(8476):307–10. 2868172

[42] Bland JM, Altman DG. Comparing methods of measurement: why plotting difference against standard method is misleading. Lancet. 1995;346(8982):1085–7. 10.1016/s0140-6736(95)91748-9 7564793

[43] Higgins JPT, Altman DG, Sterne JAC. Assessing risk of bias in included studies In: Higgins JPT, Green S, editors. Cochrane handbook for systematic reviews of interventions. Version 5.1.0. London: Cochrane Collaboration; 2011.

[44] Vale CL, Tierney JF, Burdett S. Can trial quality be reliably assessed from published reports of cancer trials: evaluation of risk of bias assessments in systematic reviews. BMJ. 2013;346:f1798 10.1136/bmj.f1798 23610376

[45] Michiels S, Piedbois P, Burdett S, Syz N, Stewart L, Pignon JP. Meta-analysis when only the median survival times are known: a comparison with individual patient data results. Int J Technol Assess Health Care. 2005;21(1):119–25. 10.1017/s0266462305050154 15736523

[46] Pogue JM, Yusuf S. Cumulating evidence from randomized trials: utilizing sequential monitoring boundaries for cumulative meta-analysis. Control Clin Trials. 1997;18(6):580–93. 10.1016/s0197-2456(97)00051-2 9408720

[47] Wetterslev J, Thorlund K, Brok J, Gluud C. Trial sequential analysis may establish when firm evidence is reached in cumulative meta-analysis. J Clin Epidemiol. 2008;61(1):64–75. 10.1016/j.jclinepi.2007.03.013 18083463

[48] Vale CL, Burdett S, Rydzewska LH, Albiges L, Clarke NW, Fisher D, et al Addition of docetaxel or bisphosphonates to standard of care in men with localised or metastatic, hormone-sensitive prostate cancer: a systematic review and meta-analyses of aggregate data. Lancet Oncol. 2016;17(2):243–56. 10.1016/S1470-2045(15)00489-1 26718929 PMC4737894

[49] Tierney JF, Vale CL, Burdett S, Fisher D, Rydzewska LHM, Parmar MKB. Timely and reliable evaluation of the effects of interventions: a framework for adaptive meta-analysis (FAME). Trials. 2017;18(Suppl 1):P351.

[50] Rydzewska LHM, Burdett S, Vale CL, Clarke NW, Fizazi K, Kheoh T, et al Adding abiraterone to androgen deprivation therapy in men with metastatic hormone-sensitive prostate cancer: a systematic review and meta-analysis. Eur J Cancer. 2017;84:88–101. 10.1016/j.ejca.2017.07.003 28800492 PMC5630199

[51] Burdett S, Boeve LM, Ingleby FC, Fisher DJ, Rydzewska LH, Vale CL, et al Prostate radiotherapy for metastatic hormone-sensitive prostate cancer: a STOPCAP systematic review and meta-analysis. Eur Urol. 2019;76(1):115–24. 10.1016/j.eururo.2019.02.003 30826218 PMC6575150

[52] Roberts I, Ker K, Edwards P, Beecher D, Manno D, Sydenham E. The knowledge system underpinning healthcare is not fit for purpose and must change. BMJ. 2015;350:h2463 10.1136/bmj.h2463 26041754

[53] Smith CT, Oyee J, Marcucci M, Rovers M, Iorio A, Riley R, et al Individual participant data meta-analyses compared with meta-analyses based on aggregate data. Trials. 2011;12(Suppl 1):A57. 10.1002/14651858.MR000007.pub3 PMC7125394 27595791

[54] Hirooka T, Hamada C, Yoshimura I. A note on estimating treatment effect for time-to-event data in a literature-based meta-analysis. Methods Inf Med. 2009;48(2):104–12. 10.3414/ME0535 19283306

[55] Guyot P, Ades AE, Ouwens MJ, Welton NJ. Enhanced secondary analysis of survival data: reconstructing the data from published Kaplan-Meier survival curves. BMC Med Res Methodol. 2012;12:9 10.1186/1471-2288-12-9 22297116 PMC3313891

[56] Wei Y, Royston P, Tierney JF, Parmar MK. Meta-analysis of time-to-event outcomes from randomized trials using restricted mean survival time: application to individual participant data. Stat Med. 2015;34(21):2881–98. 10.1002/sim.6556 26099573 PMC5695659

[57] Wei Y, Royston P, Tierney J, Parmar M. The feasibility and reliability of using restricted mean survival time in aggregate data meta-analysis of time-to-event outcomes. Abstracts of the 21st Cochrane Colloquium, Québec City, Canada. Cochrane Database Syst Rev. 2013;(9 Suppl):P3.044.

[58] Kirkham JJ, Dwan KM, Altman DG, Gamble C, Dodd S, Smyth R, et al The impact of outcome reporting bias in randomised controlled trials on a cohort of systematic reviews. BMJ. 2010;340:c365 10.1136/bmj.c365 20156912

[59] Altman DG. Better reporting of randomised controlled trials: the CONSORT statement. BMJ. 1996;313:570–1. 10.1136/bmj.313.7057.570 8806240 PMC2352018

[60] Moher D, Schulz KF, Altman D, CONSORT Group. The CONSORT Statement: revised recommendations for improving the quality of reports of parallel-group randomized trials. JAMA. 2001;285:1987–91. 10.1001/jama.285.15.1987 11308435

