# Supplementary material for: Comparison of aggregate and individual participant data approaches to meta-analysis of randomised trials: An observational study
Source: PLoS Med. 2020 Jan 31;17(1):e1003019. doi: 10.1371/journal.pmed.1003019 (PMC6993967; doi:10.1371/journal.pmed.1003019)
Supplement: S1 Checklist — (DOCX) [file pmed.1003019.s001.docx]

STROBE Statement—Checklist of items that should be included in reports of ***cohort studies***

|  | Item No | Recommendation | Page No |
| --- | --- | --- | --- |
| **Title and abstract** | 1 | (*a*) Indicate the study’s design with a commonly used term in the title or the abstract  Given explicitly in title, and indirectly in abstract |  |
|  |  | (*b*) Provide in the abstract an informative and balanced summary of what was done and what was found  Abstract section |  |
| Introduction | | | |
| Background/rationale | 2 | Explain the scientific background and rationale for the investigation being reported  Introduction section |  |
| Objectives | 3 | State specific objectives, including any prespecified hypotheses  End of Introduction section. We did not prespecify a hypothesis; rather, our objective was to investigate systemically whether and how AD and IPD results differ, with reference to a specific cohort of trials and reviews. |  |
| Methods | | | |
| Study design | 4 | Present key elements of study design early in the paper  Methods section, para 1 |  |
| Setting | 5 | Describe the setting, locations, and relevant dates, including periods of recruitment, exposure, follow-up, and data collection  Methods section, para 1 |  |
| Participants | 6 | (*a*) Give the eligibility criteria, and the sources and methods of selection of participants. Describe methods of follow-up  Methods section, para 1 |  |
|  |  | (*b*) For matched studies, give matching criteria and number of exposed and unexposed  Not applicable; this study did not use matching |  |
| Variables | 7 | Clearly define all outcomes, exposures, predictors, potential confounders, and effect modifiers. Give diagnostic criteria, if applicable  Outcomes and exposures: Methods section, para 2  Confounders and effect modifiers: Methods section, paras 8 and 9 |  |
| Data sources/ measurement | 8* | For each variable of interest, give sources of data and details of methods of assessment (measurement). Describe comparability of assessment methods if there is more than one group  Methods section, para 3 |  |
| Bias | 9 | Describe any efforts to address potential sources of bias  Methods section, paras 4 and 9 |  |
| Study size | 10 | Explain how the study size was arrived at  As described in para 1 of Methods section, the study size was determined simply with reference to the number of reviews completed by the Meta-analysis Group of the MRC Clinical Trials Unit at UCL over a 19-year period. No comparable additional reviews were available. |  |
| Quantitative variables | 11 | Explain how quantitative variables were handled in the analyses. If applicable, describe which groupings were chosen and why  We used raw numbers and proportions of trials, patients and events. We did not categorise, but we did log-transform proportions to better approximate normality (see Methods section, para 8). |  |
| Statistical methods | 12 | (*a*) Describe all statistical methods, including those used to control for confounding |  |
|  |  | (*b*) Describe any methods used to examine subgroups and interactions |  |
|  |  | (*c*) Explain how missing data were addressed |  |
|  |  | (*d*) If applicable, explain how loss to follow-up was addressed |  |
|  |  | (*e*) Describe any sensitivity analyses  (a) Methods section, paras 7 and 8  (b) Methods section, para 7  (c) and (d) are not applicable to this study  (e) Methods section, para 9 |  |
| Results | | |  |
| Participants | 13* | (a) Report numbers of individuals at each stage of study—eg numbers potentially eligible, examined for eligibility, confirmed eligible, included in the study, completing follow-up, and analysed |  |
|  |  | (b) Give reasons for non-participation at each stage |  |
|  |  | (c) Consider use of a flow diagram  Results section, para 1; Table 1. A flow diagram was not considered necessary, as the cohort is self-contained. |  |
| Descriptive data | 14* | (a) Give characteristics of study participants (eg demographic, clinical, social) and information on exposures and potential confounders |  |
|  |  | (b) Indicate number of participants with missing data for each variable of interest |  |
|  |  | (c) Summarise follow-up time (eg, average and total amount)  Note: “study participants” are the trials or reviews in the context of this study.  Descriptions are given in para 1 of Results section, and in Table 1. |  |
| Outcome data | 15* | Report numbers of outcome events or summary measures over time  Not applicable to this study. The outcome summary measures were the outcomes reported by the trials and reviews at publication, or as calculated from supplied IPD. |  |

| Main results | 16 | (*a*) Give unadjusted estimates and, if applicable, confounder-adjusted estimates and their precision (eg, 95% confidence interval). Make clear which confounders were adjusted for and why they were included |  |
| --- | --- | --- | --- |
|  |  | (*b*) Report category boundaries when continuous variables were categorized |  |
|  |  | (*c*) If relevant, consider translating estimates of relative risk into absolute risk for a meaningful time period  (a): Results section, paras 3 and 7  (b) and (c) Not applicable to this study |  |
| Other analyses | 17 | Report other analyses done—eg analyses of subgroups and interactions, and sensitivity analyses  Results section, para 8; Web Table 1 |  |
| Discussion | | | |
| Key results | 18 | Summarise key results with reference to study objectives  Discussion, para 1 |  |
| Limitations | 19 | Discuss limitations of the study, taking into account sources of potential bias or imprecision. Discuss both direction and magnitude of any potential bias  Discussion, para 3 |  |
| Interpretation | 20 | Give a cautious overall interpretation of results considering objectives, limitations, multiplicity of analyses, results from similar studies, and other relevant evidence  Discussion, para 6 |  |
| Generalisability | 21 | Discuss the generalisability (external validity) of the study results  Discussion, paras 2 & 3 |  |
| Other information | | | |
| Funding | 22 | Give the source of funding and the role of the funders for the present study and, if applicable, for the original study on which the present article is based  See Competing interests, Author Contributions and Role of the Sponsor.  Note that all of the meta-analyses in the cohort were originally carried out using funding from the Medical Research Council. |  |

*Give information separately for exposed and unexposed groups.

**Note:** An Explanation and Elaboration article discusses each checklist item and gives methodological background and published examples of transparent reporting. The STROBE checklist is best used in conjunction with this article (freely available on the Web sites of PLoS Medicine at http://www.plosmedicine.org/, Annals of Internal Medicine at http://www.annals.org/, and Epidemiology at http://www.epidem.com/). Information on the STROBE Initiative is available at http://www.strobe-statement.org.
